# An Aggressive Case of Thymic Lymphoepithelial Carcinoma Complicated by Cytokine Release Syndrome: A Case Report

**DOI:** 10.1155/crom/4842642

**Published:** 2025-12-17

**Authors:** Louis G. Filipiak, Nyembezi L. Dhliwayo, Koosha Paydary

**Affiliations:** ^1^ Rush University Medical Center, Chicago, Illinois, USA, rush.edu

## Abstract

Thymic lymphoepithelial carcinomas (TLECs) are a rare primary thymic cancer best described by lymphoplasmacytic infiltration of the thymic stroma. Though only accounting for 1.3%–6% of thymic carcinomas, the distribution of TLECs is bimodal, peaking around ages 14 and 48 and affecting males to females in a 2:1 ratio. Due to their rarity and aggressive nature, they often present at later stages and can be primarily treated with a combination of chemotherapy and radiation. While several paraneoplastic syndromes have been reported with TLECs, cytokine release syndrome (CRS) has been observed in several cases. This particular case illustrates a metastatic TLEC patient that was treated with chemotherapy and complicated by CRS.

## 1. Introduction

Thymic lymphoproliferative carcinoma (TLEC) is a rare primary thymic cancer with significant undifferentiated reactive lymphoplasmacytic infiltration [1]. Histologically, TLEC may appear identical to nasopharyngeal carcinomas and is highly associated with the Epstein–Barr virus (EBV), which is positive in 52.2% of cases, according to a previous systematic review [1, 2]. TLECs account for around 14% of thymic epithelial tumors and 1.3%–6% of thymic carcinomas, the remainder of which do not have an association with EBV. TLECs often metastasize to the liver, lung, and bone and consequently have a poor prognosis, though this is based on case reports alone due to the disease′s rarity [1, 2]. In one literature review of 58 cases that were reported, the median survival time was 22 months with a 5‐year survival rate of 34.4%. Of note, the prognosis of TLECs is not affected by the presence or absence of EBV. The age distribution of those who present with TLEC is bimodal, with two peaks around ages 14 and 48 with a median age of 41. TLECs appear to affect males over females in a 2:1 ratio, and there have not been any geographical differences found in presentations across Asia, Europe, and the United States [1].

Moreover, cytokine release syndrome (CRS) is a potentially life‐threatening toxicity that has been observed with the burgeoning use of both natural and artificial antibodies in cancer therapy [3]. While CRS can stem from lymphoproliferative disorders like TLEC, it is often seen as a result of adoptive T‐cell therapies such as bispecific antibody therapy or CAR‐T therapies. CRS is a nonspecific immune activation that, in the setting of leukoproliferative disorders such as leukemias or lymphomas, can cause a massive immune response and toxicity via the release of inflammatory cytokines such as IL‐6, TNF‐a, and IFN‐g [3]. The common presentation is akin to that of a septic shock picture: fever, tachycardia, hypotension, tachypnea, and fatigue, as well as skin rashes, nausea, vomiting, and diarrhea. Downstream effects include acute kidney injury and transaminitis. The timing of onset for CRS can be minutes to hours after administration of a T‐cell activating agent, though timing is more difficult to predict in naturally occurring lymphoproliferative disorders. Diagnosis of CRS is clinical, though it is usually related to bispecific antibody therapy, CAR‐T therapy, or lymphoproliferative disorders and is dependent on the level of response. Some indicators of CRS, however, include nonspecific inflammatory markers such as ESR, CRP, and IL‐6. Treatment involves supportive care as well as immunosuppressive agents such as corticosteroids, though tocilizumab can play a role in moderate to severe disease. As the mechanism of tocilizumab is that of an IL‐6 receptor antibody, it is able to bind and neutralize the deleterious inflammatory effects of IL‐6 in the cytokine proliferation pathway [4]. Of note, the incidence and severity of CRS are often seen in patients with a higher tumor burden as greater T‐cell activation often occurs. This case reports a patient with metastatic TLEC that was initially treated with chemotherapy with a course complicated by CRS.

## 2. Case

**Figure 1:**
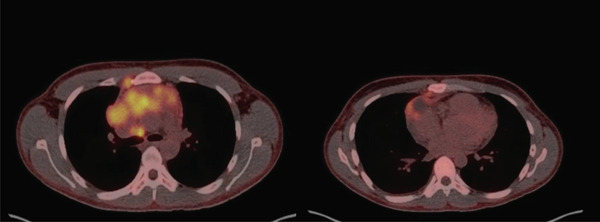
Initial PET‐CT at diagnosis showing a large lobulated 10.2 × 4 cm lobulated FDG‐avid anterior mediastinal mass, loss of fat planes between the mass and major vessels, SUV max 8.5; mass extending into the mediastinum and thoracic inlet. Also with mildly PET‐avid bilateral pleural effusion and pericardial effusion; bilateral paratracheal, anterior jugular, lower jugular lymphadenopathy, anterior pericardiac/diaphragmatic, and right internal mammary lymphadenopathy. T5, L1, and left posterior eighth rib osseous metastasis also evident [5].

A 19‐year‐old male with no major past medical history presented to the emergency room with 4 months of bilateral shoulder and neck pain. While at the ED, a plain chest radiograph showed a widening of the mediastinum with concern for a possible mass. A CT chest was subsequently performed, showing a 9.9 cm mass with severe narrowing of the superior vena cava and left brachiocephalic vein as well as nonspecific mediastinal lymphadenopathy. A 7‐mm pulmonary nodule was also noted. Interventional radiology was consulted for diagnostic sampling of the mass. Core biopsies of the mediastinal mass were done, with surgical pathology significant for a thymic lymphoepithelial carcinoma. Of note, the biopsy immunohistochemistry was positive for CK8/18, p63, CK5, and CD5. Due to this result, an MRI of the chest was performed which was positive not only for the mediastinal mass but also for enhancing lesions in T7 and L1 concerning for metastases. As shown in Figure 1, his staging PET‐CT scan showed the mass as well as multiple hypermetabolic mediastinal, supraclavicular, internal mammary, right cardiophrenic, and diaphragmatic lymph nodes concerning for metastases, along with the spinal lesions. Unfortunately, after the biopsy was taken, the patient developed persistent tachycardia and dyspnea on exertion, likely due to the near‐complete encasement of the superior vena cava. The patient was seen by the rheumatology team during this time, who found that he was also positive for ANA, anti‐dsDNA, and RF antibodies. The patient was consequently given the diagnosis of paraneoplastic polyarthritis. The patient was discharged on dexamethasone 4 mg every 8 h and oxycodone for pain. Finally, of note, the patient was found based on a chart review to have high EBV titers.

**Figure 2:**
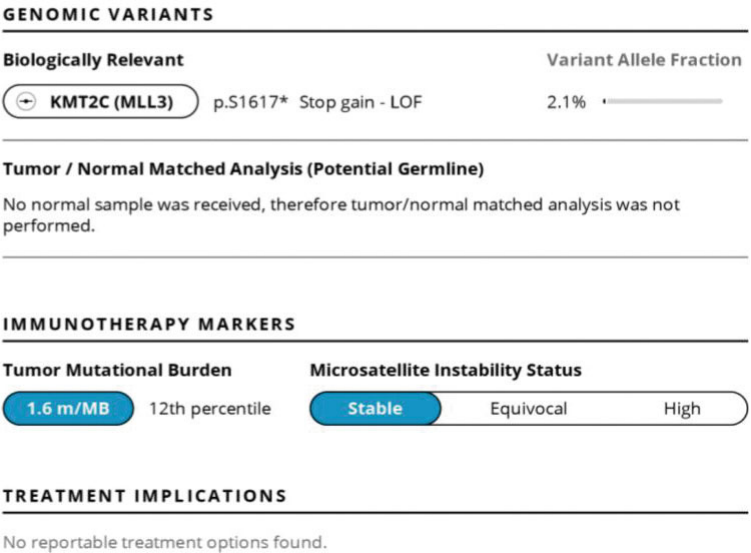
NeoGenomics sequencing with Tempus xT assay showing KMT2C stop gain loss of function with a low variable allelic frequency (2.1%), but no actionable mutation variant; low tumor mutational burden and stable microsatellite instability status [5].

**Figure 3:**
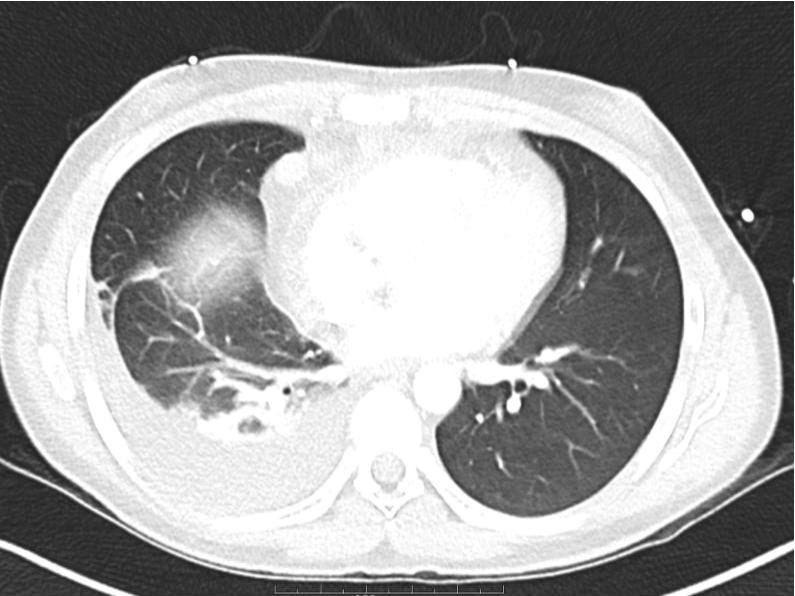
CT chest with angiography: interval increase in anterosuperior mediastinal mass to 12.3 × 9.9 cm with SVC compression; progressive pericardial, moderate right effusion, and lower lobe consolidation [5].

**Figure 4:**
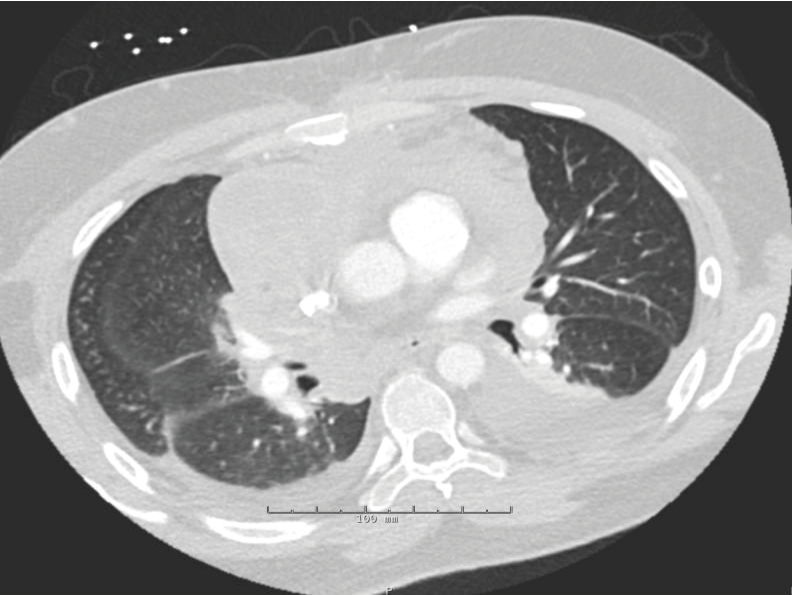
CT chest with angiography: stable anterior mediastinal mass to 6.1 × 5.3 cm (previously noted to decrease in size). Partial bilateral lower lobar atelectasis, interlobular septal thickening increased right anterior and right anterior mammary lymph node, anterior diaphragmatic and anterior mediastinal lymphadenopathy. Increased moderate bilateral pleural effusions [5].

The patient′s case was discussed in a multidisciplinary thoracic tumor board; due to the metastatic nature of his disease, his thymic carcinoma was deemed unresectable. As shown in Figure 2, next‐generation sequencing (NGS) testing was performed on this patient, which showed a nonactionable KMT2C mutation as well as a tumor mutational burden of 1.6. The patient was subsequently started on carboplatin and paclitaxel for his chemotherapeutic regimen, though pembrolizumab, which was supposed to be a component of his initial regimen, was delayed due to insurance issues. Unfortunately, after the first cycle, the patient developed fever, diarrhea, and syncope due to hypovolemia, indicating inpatient admission for aggressive resuscitation. While antibiotics were initially started, the infectious workup was unremarkable, and the patient was eventually taken off antibiotics. He was then started on prednisone 30 mg BID due to concern for CRS. This improved his fever and diarrhea, and he was eventually discharged. During this time, his mediastinal mass unfortunately increased in size as seen on CT angiography of the chest in Figure 3. He consequently continued onto Cycle 2 of carboplatin and now dose‐reduced paclitaxel due to peripheral neuropathy. A follow‐up CT scan of the chest, abdomen, and pelvis was performed, which showed a stable mediastinal mass and decreasing pulmonary nodule but increasing anterior mediastinal lymph nodes as seen in Figure 4.

The patient was then readmitted for similar fever and tachycardia, but also hyponatremia. His IL‐6, ESR, CRP, and ferritin levels were all elevated, and he was subsequently started on stress‐dose methylprednisolone for concern for CRS once again. The oncology team further recommended two doses of tocilizumab 540 mg, and the patient eventually improved and was transitioned to a dexamethasone taper. After discharge, given his multiple episodes of CRS, systemic therapy was transitioned to lenvatinib 24 mg daily. This was complicated by cutaneous toxicity, including multiple bullous ulcers on his left upper chest, lower abdomen, and bilateral axillae.

The patient was subsequently admitted for acute onset neck pain, swelling, and dysphagia, concerning for mediastinitis, with imaging notable for a retropharyngeal collection. Though his airway was patent on imaging, interventional radiology placed a stent to decompress the superior vena cava. His course was further complicated by respiratory decompensation, needing high‐flow oxygen supplementation; he was found to have acute left internal jugular venous thromboembolism.

Given aggressive disease, intractable pain, and moderate toxicities from systemic therapy, as well as tenuous respiratory status, the patient′s family elected comfort‐focused care. The patient was ultimately discharged home with supportive hospice care.

## 3. Discussion

As posited, this patient was a male close to Age 19, who presented with nonspecific symptoms, and was found to be in the later stages of TLEC upon initial imaging studies. Histologically, TLECs present with significant lymphocyte and plasma cell infiltration into the thymic stroma. Germinal cells, eosinophils, and granulomas can also be present. Immunohistochemical staining often shows tumor cells positive for cytokeratin, specifically CK19, CK8, and CK18 which are often poor prognostic markers in other squamous cell cancers. TLECs can also stain positive for CD5, p63, and occasionally CD117. Lastly, the Ki‐67 index in TLECs is often reported to be high, indicating rapidly dividing and highly malignant cells within this cancer type [1].

TLEC patients often initially present with nonspecific symptoms such as chest pain, cough, fatigue, and dyspnea and are usually discovered via imaging at later stages. However, they can also be discovered in asymptomatic patients during routine imaging studies. TLECs often spread into surrounding tissues such as the lungs, heart, diaphragm, and major vessels such as the superior vena cava, often causing obstruction. Rare paraneoplastic syndromes have been associated with TLEC, such as hypertrophic osteoarthropathy, polymyositis, arthropathy, and systemic lupus erythematosus (SLE) [1]. There have been no reports of classic thymoma paraneoplastic syndromes such as myasthenia gravis, pure red cell aplasia, or hypogammaglobulinemia in TLECs. Treatment involves a combination of surgery, radiation, and chemotherapy [2]. According to NCCN guidelines, first‐line therapy for locally advanced unresectable metastatic carcinoma involves both radiation and carboplatin and paclitaxel, though ramucirumab can be added in some cases. Second‐line therapy can include combination gemcitabine and capecitabine or tyrosine kinase inhibitors like lenvatinib or sunitinib [6]. While immunotherapy agents can be used for thymic carcinomas, they are often reserved for at least second‐line therapy due to a higher rate of immune‐mediated adverse events. Of note, one case review of 40 patients showed that those who received surgical resection had a 5‐year survival rate of 50.2% versus 11.2% in those who did not (*p* = 0.0029).

Due to the significant tumor burden, the patient had near obstruction of the superior vena cava. He also presented with paraneoplastic polyarthropathy which was treated with corticosteroids. While he initially was started on a regimen of carboplatin and paclitaxel, he transitioned to the tyrosine kinase inhibitor lenvatinib due to complications. These complications included CRS, which was also originally treated with corticosteroids before shifting to tocilizumab therapy. Unfortunately, similar to the general prognosis of TLEC patients, this patient eventually elected to be discharged home with supportive hospice care.

## Consent

Verbal consent was obtained from the patient. No written consent was obtained from the patient as there is no patient‐identifiable data included in this case report. Patient information is sufficiently anonymized per the ICMJE guidelines.

## Disclosure

The authors have nothing to report.

## Conflicts of Interest

The authors declare no conflicts of interest.

## Funding

No funding was received for this manuscript.

## Data Availability

The data that support the findings of this study are available from the corresponding author upon reasonable request.
